# Rationale and design of a multicenter randomized study for evaluating vascular function under uric acid control using the xanthine oxidase inhibitor, febuxostat: the PRIZE study

**DOI:** 10.1186/s12933-016-0409-2

**Published:** 2016-06-18

**Authors:** Jun-ichi Oyama, Atsushi Tanaka, Yasunori Sato, Hirofumi Tomiyama, Masataka Sata, Tomoko Ishizu, Isao Taguchi, Takanori Kuroyanagi, Hiroki Teragawa, Nobukazu Ishizaka, Yumiko Kanzaki, Mitsuru Ohishi, Kazuo Eguchi, Yukihito Higashi, Hirotsugu Yamada, Koji Maemura, Junya Ako, Yasuko K. Bando, Shinichiro Ueda, Teruo Inoue, Toyoaki Murohara, Koichi Node, On behalf of the PRIZE Study Investigators

**Affiliations:** Department of Cardiovascular Medicine, Saga University Faculty of Medicine, 5-1-1 Nabeshima, Saga, 849-8501 Japan; Department of Clinical Research, Chiba University Graduate School of Medicine, Chiba, Japan; Department of Cardiology, Tokyo Medical University, Tokyo, Japan; Department of Cardiovascular Medicine, Institute of Biomedical Sciences, Tokushima University Graduate School, Tokushima, Japan; Department of Clinical Laboratory Medicine, Faculty of Medicine, University of Tsukuba, Tsukuba, Japan; Department of Cardiology, Dokkyo Medical University Koshigaya Hospital, Koshigaya, Japan; Department of Cardiovascular Medicine, Hiroshima General Hospital of West Japan Railway Company, Hiroshima, Japan; Internal Medicine (III), Department of Cardiology, Osaka Medical College, Takatsuki, Japan; Department of Cardiovascular Medicine and Hypertension, Kagoshima University, Kagoshima, Japan; Division of Cardiovascular Medicine, Department of Medicine, Jichi Medical University School of Medicine, Shimotsuke, Japan; Department of Cardiovascular Regeneration and Medicine, Research Institute for Radiation Biology and Medicine, Hiroshima University, Hiroshima, Japan; Department of Cardiovascular Medicine, Nagasaki University Graduate School of Biomedical Sciences, Nagasaki, Japan; Department of Cardiovascular Medicine, Kitasato University, Sagamihara, Japan; Department of Cardiology, Nagoya University Graduate School of Medicine, Nagoya, Japan; Department of Clinical Pharmacology & Therapeutics, University of the Ryukyus, Nishihara, Japan; Department of Cardiovascular Medicine, Dokkyo Medical University, Mibu, Japan

**Keywords:** Carotid artery, Febuxostat, Hyperuricemia, Intima-media thickness (IMT), Randomized controlled trial, Xanthine oxidase inhibitor

## Abstract

**Background:**

Xanthine oxidase inhibitors are anti-hyperuricemic drugs that decrease serum uric acid levels by inhibiting its synthesis. Xanthine oxidase is also recognized as a pivotal enzyme in the production of oxidative stress. Excess oxidative stress induces endothelial dysfunction and inflammatory reactions in vascular systems, leading to atherosclerosis. Many experimental studies have suggested that xanthine oxidase inhibitors have anti-atherosclerotic effects by decreasing in vitro and in vivo oxidative stress. However, there is only limited evidence on the clinical implications of xanthine oxidase inhibitors on atherosclerotic cardiovascular disease in patients with hyperuricemia. We designed the PRIZE study to evaluate the effects of febuxostat on a surrogate marker of cardiovascular disease risk, ultrasonography-based intima-media thickness of the carotid artery in patients with hyperuricemia.

**Methods:**

The study is a multicenter, prospective, randomized, open-label and blinded-endpoint evaluation (PROBE) design. A total of 500 patients with asymptomatic hyperuricemia (uric acid >7.0 mg/dL) and carotid intima-media thickness ≥1.1 mm will be randomized centrally to receive either febuxostat (10–60 mg/day) or non-pharmacological treatment. Randomization is carried out using the dynamic allocation method stratified according to age (<65, ≥65 year), gender, presence or absence of diabetes mellitus, serum uric acid (<8.0, ≥8.0 mg/dL), and carotid intima-media thickness (<1.3, ≥1.3 mm). In addition to administering the study drug, we will also direct lifestyle modification in all participants, including advice on control of body weight, sleep, exercise and healthy diet. Carotid intima-media thickness will be evaluated using ultrasonography performed by skilled technicians at a central laboratory. Follow-up will be continued for 24 months. The primary endpoint is percentage change in mean intima-media thickness of the common carotid artery 24 months after baseline, measured by carotid ultrasound imaging.

**Conclusions:**

PRIZE will be the first study to provide important data on the effects of febuxostat on atherosclerosis in patients with asymptomatic hyperuricemia.

*Trial Registration* Unique trial Number, UMIN000012911 (https://upload.umin.ac.jp/cgi-open-bin/ctr/ctr.cgi?function=brows&action=brows&type=summary&recptno=R000015081&language=E)

**Electronic supplementary material:**

The online version of this article (doi:10.1186/s12933-016-0409-2) contains supplementary material, which is available to authorized users.

## Background

Uric acid (UA) is an end product of the purine metabolic pathway catalyzed by xanthine oxidase (XO), with increased levels of UA associated closely with various pathophysiologies [1, 2]. Evidence from clinical studies has suggested that hyperuricemia is associated with the characteristics of the metabolic syndrome (MetS), including obesity, hypertension (HT), hyperlipidemia (HL), diabetes mellitus (DM) [3–7], and cardiovascular disease (CVD) [8–11]. Takayama et al. [12] showed in both males and females without the MetS that hyperuricemia is an independent risk factor for the incidence of carotid atherosclerosis imaged by ultrasound. Kawamoto et al. [13] also demonstrated that the prevalence of the MetS increased according to serum UA levels only in women and in men without the Mets, suggesting that UA levels were an independent risk factor of carotid atherosclerosis. Although UA is considered to be a risk factor or marker for CVD [14, 15], there is still some controversy regarding its independent contribution to CVD risk. For example, it has been reported that there is no relationship between UA levels and the increased risk of death from all causes, including CVD and stroke in Japanese people aged ≥30 years [16].

Although the mechanisms for the effects of hyperuricemia on atherosclerosis remain uncertain [17, 18], possible pathophysiological actions may include several adverse effects on endothelial dysfunction, oxidative metabolism, inflammation, and platelet adhesiveness and aggregation [19–22]. The mediators and cytokines produced by these processes interact with background pathology such as the MetS and insulin resistance, leading to cellular damage. Recent studies in animal models report that UA may have a causal role in the development of atherosclerosis, whereas lowering uric acid levels prevented or reversed the features of the MetS [23].

Febuxostat, a novel non-purine selective inhibitor of XO was developed 40 years ago and was the first XO inhibitor with stronger UA-lowering effects [24]. Compared to allopurinol, a conventional XO inhibitor, febuxostat can be administered to patients with mild to moderately impaired renal function because of its dual excretion pathway [24]. Recent reports support the effectiveness of febuxostat in the treatment of patients with gout [25, 26]. Febuxostat is now used widely in hyperuricemia patients both with and without relevant complications. A randomized trial on post-cardiac surgery patients with hyperuricemia showed that febuxostat had superior anti-inflammatory and renoprotective effects than allopurinol [27]. In experimental studies, XO inhibition by febuxostat reduced the production of reactive oxygen species (ROS) to a greater extent than allopurinol and attenuated experimental atherosclerosis in mice [28–30]. However, there is only limited evidence on the effect of pharmacological intervention on carotid atherosclerosis in patients with hyperuricemia.

The PRIZE study was designed with the aim to evaluate the effects of febuxostat on atherosclerosis in patients with hyperuricemia by measuring changes in carotid intima-media thickness (IMT), a marker of atherosclerosis. This study will be the first to provide clinical evidence as to whether or not an anti-hyperuricemic agent, febuxostat, can suppress carotid atherosclerosis.

## Methods

### Study overview and design

The PRIZE study is a multicenter, prospective, randomized, open-label, blinded-endpoint (PROBE) clinical trial that is being carried out in Japan. The study aims to test the hypothesis that febuxostat treatment is superior to non-pharmacological treatment for hyperuricemia to prevent progression of carotid atherosclerosis, measured as IMT, after 24 months of treatment. Eligible patients are assigned randomly (ratio 1:1) to either a febuxostat group (10–60 mg/day) or a control group (non-pharmacological treatment). Because the usual daily dosage of febuxostat is 10–60 mg in Japan, participants assigned to the febuxostat group receive an initial dose of 10 mg/day that can be subsequently increased to 60 mg/day to achieve an appropriate UA level. The study protocol states that all the patients will be followed up annually for 24 months.

Prior to initiation, the study protocol was approved by the local institutional review board and independent ethics committee at every site. The trial will be conducted in full compliance with the Declaration of Helsinki and according to the Ethical Guidelines for Medical and Health Research Involving Human Subjects established by the Ministry of Health, Labour, and Welfare and Ministry of Education, Culture, Sports, Science, and Technology.

### Study population

A total of 500 patients with hyperuricemia are scheduled to be enrolled between 2014 and 2016 and then followed-up for 24 months. Prior to assessment of eligibility, every patient is required to receive an adequate explanation of the study plan, with written informed consent then obtained from each patient. The inclusion and exclusion criteria for the study are listed in Table 1. In brief, these criteria include: eligible patients aged ≥20 years with asymptomatic hyperuricemia (UA >7.0 mg/dL) and a maximum carotid IMT ≥1.1 mm. A UA level of 7.0 mg/dL is defined as the minimal level for diagnosing hyperuricemia in Japan, with medical intervention in such patients being considered valid [31]. Patients who have received any UA-lowering agents within the 8-week period prior to enrollment are not enrolled in the study.

**Table 1:** Detailed inclusion and exclusion criteria

Inclusion	Exclusion
Adults (aged ≥20 years) Patients with asymptomatic hyperuricemia with a serum UA >7.0 mg/dL Patients with a maximum IMT ≥1.1 mm The patient provided written informed consent to participate in the study	Patients being treated with any of the following antihyperuricemic agents within 8 weeks before confirmation of the eligibility criteria: allopurinol, benzbromarone, probenecid, bucolome, topiroxostat, or febuxostat Patients being treated with any of the following agents at the time of confirmation of the eligibility criteria: mercaptopurine hydrate, azathioprine, vidarabine, or didanosine Patients who have undergone an operation or who have severe infections or serious injury at the time of confirmation of the eligibility criteria. Patients who had a myocardial infarction, angina pectoris, percutaneous transluminal coronary angioplasty/bypass surgery, cerebral infarction, cerebral hemorrhage, subarachnoid hemorrhage, or transient cerebral ischemic attack within 3 months before confirmation of the eligibility criteria Patients with cardiac dysfunction (NYHA class IV) Patients with gouty tophus, or those who have subjective symptoms of gout arthritis within 1 year before confirmation of the eligibility criteria Patients with a complication or a disease history (eGFR <30 mL/min/1.73 m^2^ or patients on dialysis) Patients with severe liver dysfunction (AST or ALT ≥2 times the upper limit of the institutional standard value) Patients with a complication or a disease history (e.g. malignant tumor) who are considered not eligible for the study by the attending doctor Patients with a history of hypersensitivity to febuxostat Pregnant, possibly pregnant, or lactating women or those who wish to become pregnant during participation in the study Patients who have undergone CEA or CAS surgery Patients who are considered not eligible for the study by the attending doctor due to other reasons

*ALT* alanine aminotransferase, *AST* aspartate transaminase, *CAS* carotid artery stenting, *CEA* carotid endarterectomy, *eGFR* estimated glomerular filtration rate, *IMT* intima-media thickness, *NYHA* New York Heart Association, *UA* uric acid

### Randomization

Randomization takes place following an initial ultrasonographic estimation of carotid IMT at the PRIZE Data Center. Randomization is performed using a modified minimization method with a biased-coin assignment balanced for age (<65, ≥65 years), gender, presence or absence of type 2 DM, UA (<8, ≥8 %), and maximum IMT (<1.3, ≥1.3 mm). Random allocation incorporating a stratified technique is generated automatically using a minimization method on a computer program [32].

### Treatment outline

**Fig. 1:**
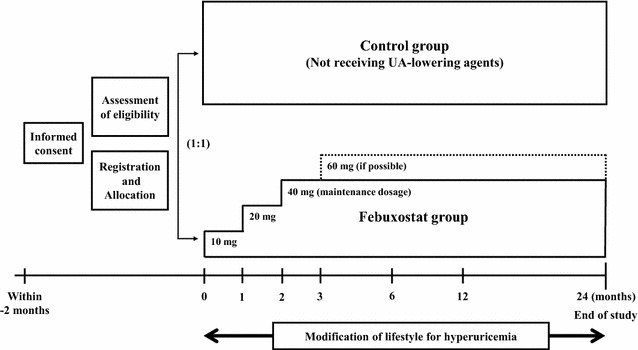
Study outline

All participants in both groups need to receive and continue an appropriate diet (Fig. 1) and exercise therapy for hyperuricemia, using the treatment brochure for the current study modified from the treatment guideline [31]. Patients assigned to the febuxostat group receive an initial dose of 10 mg/day that is increased to 20 mg/day at 1 month and 40 mg/day at 2 months. Febuxostat 40 mg daily is the principle maintenance dosage up to 24 months, but at 12 months or later the dose of febuxostat is increased to 60 mg/day, if possible. If UA levels decrease to ≤2.0 mg/dL during the study period, the next incremental step of febuxostat dose will not be needed and the dose is decreased to the preceding step. On the other hand, if the UA level is >12.0 mg/dL or gouty arthritis develops as an adverse event, the investigators must discontinue the study treatment and initiate appropriate procedures and treatments. The details of the discontinuance criteria are listed in Table 2. Drugs that must not be used are allopurinol, benzbromarone, probenecid, bucolome, and topiroxostat in both groups, and febuxostat in the control group. The participant’s background treatment, such as anti-diabetic agents, antiplatelet agents, anti-hypertensive agents, and lipid-lowering agents remains unchanged, if possible, during the study period, taking into account the appropriate clinical severity of the diseases.

**Table 2:** Discontinuance criteria

Serum UA >12.0 mg/dL
Onset of gout arthritis
Considered inappropriate to continue the study by investigators due to adverse side effects or serious adverse events
Needed to receive any prohibited concomitant drugs
Participant relocated or changed doctor
The participant decides they no longer wish to continue the study
Considered inappropriate to continue the study by investigators due to some other reason

*UA* uric acid

### Measurement of carotid IMT

The initial carotid ultrasound examinations are performed at each site to determine the eligibility prior to study inclusion and then measured at a core laboratory (Tsukuba University) at 0, 12, and 24 months after randomization. High-resolution carotid ultrasonography is performed at each ultrasound laboratory using standardized imaging protocols and systems equipped with >7.5 MHz linear transducers. Expert trained sonographers who have attended a lecture on measuring carotid IMT carry out the procedure, according to the method recommended by the Mannheim carotid IMT consensus [33, 34]. Longitudinal B-mode images, perpendicular to the ultrasound beam, with a 3–4 cm imaging depth, are recorded in the distal common carotid arteries (CCA), bulbs, and proximal internal carotid arteries (ICA) on both sides. The lateral probe incidence is used to obtain CCA images, using external landmarks with an original semicircular protractor developed for this purpose. The mean CCA-IMT indicates the average IMT value of the right and left CCA-IMT, 10 mm from the bulb. The following far wall IMTs will be measured: maximum IMT of the CCA; mean and maximum IMTs of the bulb and ICA; and the plaque area with the lowest echogenicity and its median gray scale [35]. Plaque is defined as a focal region with an intima-media thickness ≥1.1 mm that protrudes into the lumen, and is distinct from the adjacent boundary. The optimized R-wave gated still frames of the carotid IMT are stored as JPEG files, with all the parameters collected and measured at the core laboratory. An expert analyzer unaware of the clinical information of the subjects will measure all the IMT values using an automatic IMT measurement software program (Vascular Research Tools 5, Medical Imaging Applications, Iowa, USA) [36]. The analyzer will select the best images of the right and left CCA, bulb, and ICA. The software program identifies the lumen/intima and the media/adventitia borders in this region and calculates the distance between them.

### Outcomes

The primary endpoint is the percentage change in mean IMT of the common carotid artery 24 months from baseline.

The secondary endpoints are as follows: (1) Ultrasonography parameters at baseline and, after 12 and 24 months, expressed as the magnitude of change and percentage change from baseline of mean IMT, maximum IMT in the common carotid artery, bulbs, and internal artery, and plaque area and echogenicity. If multiple plaques are observed in a patient, a plaque with the lowest echogenicity at baseline will be selected for assessment of plaque area and echogenicity. (2) Serum UA levels at baseline, and after 6, 12, and 24 months, expressed as the magnitude of change and percentage change from baseline. (3) Levels of the following clinical parameters at baseline and after 6, 12, and 24 months, expressed as the magnitude of change and percentage change from baseline; serum levels of total cholesterol, low-density lipoprotein (LDL) cholesterol (indirect method), high-density lipoprotein (HDL) cholesterol, triglyceride, non-HDL cholesterol, creatinine, estimated glomerular filtration rate, urinary albumin excretion (in patients with DM), urinary liver-type fatty acid binding protein (in patients with DM or chronic kidney disease). (4) Blood pressure levels measured at a clinic and N-terminal pro-brain natriuretic peptide levels at baseline and after 12 and 24 months, expressed as the magnitude of change and percentage change from baseline. (5) Composite events of cardiovascular death, non-fatal myocardial infarction, and stroke, renal events such as doubling of serum creatinine, initiation of renal replacement therapy, or renal transplantation, and all-cause death, expressed as the incidence of each event in individuals from baseline to 24 months or at study discontinuation. (6) Adverse events that occurred between baseline and 24 months. The exploratory endpoints include values at baseline and after 12 and 24 months, and the magnitude of change and percentage change from baseline in cardiovascular-related examinations including flow-mediated dilatation (FMD), pulse wave velocity (PWV), cardio-ankle vascular index (CAVI), echocardiography (systolic and diastolic function, left atrial dimension, left ventricular mass index), and augmentation index (AI) and the following biomarkers: high-sensitivity C-reactive protein, 1.5AG, small dense LDL, remnant lipoprotein cholesterol, malondialdehyde-altered LDL, serum cystatin C, receptor for advanced glycation end products, high-molecular weight adiponectin, high-sensitive troponin I, angiopoietin-like protein 2, and blood pressure measured at home.

### Statistical considerations

#### Sample size and power calculation

We assumed the percentage changes in average carotid IMT in the control and febuxostat groups 24 months after randomization in the study would be as follows:

A percentage change in average carotid IMT of +1.96 % at 18 months based on our previous database (unpublished data). We estimated the increase from baseline would be +2.62 % after 24 months. The standard deviation (SD) will be same as that measured in our previous study. We assume that the percentage change in average carotid IMT will be +2.6 ± 6.0 % (mean ± SD) in the control group. On the other hand, there is no data about the effect of febuxostat on carotid IMT. The effect of febuxostat was set conservatively at +1.0 ± 6.0 % based on data on pitavastatin from a previous study that showed an annual rate of change in carotid IMT of −0.8 % [37]. Based on these assumptions of a 1.6 % group difference in the primary endpoint at 24 months and a SD of 6.0 % for individual differences to achieve 90 % power for a two-sided, two-sample t test at the 0.05 significance level, a sample size of 250 patients in each group is required. A total of 500 patients will be enrolled in the study in anticipation of a 10 % dropout rate.

#### Statistical analysis plan

The statistical analysis and reporting of this trial will be conducted in accordance with the CONSORT guidelines, with the primary analyses based on the intention-to-treat principle. For the baseline variables, summary statistics will be calculated and expressed as frequencies and proportions for categorical data, and means and SD for continuous variables. Baseline variables will be compared using Fisher’s exact test for categorical outcomes and unpaired t-tests for continuous variables.

For the primary analysis comparing treatment effects, the baseline-adjusted means and their 95 % confidence intervals (CIs), estimated by analysis of covariance, were compared between treatments (febuxostat group vs. the control group). This will be carried out taking into account the variation due to treatment effects, and using the allocation adjustment factors as the covariates. To compare the treatment groups, the differences in the baseline-adjusted means and associated 95 % CIs will be expressed as a proportion of the reference treatment baseline-adjusted means. The primary analysis will not impute missing observations, with the mixed effects model for repeated measures (MMRM) being used as a sensitivity analysis to examine the effect of missing data. In addition, MMRM will be used as a sensitivity analysis to examine the outcomes at months 0 and 24 modeled as a function of time, treatment, and treatment-by-time interaction. The secondary analysis will be performed in the same manner as the primary analysis.

All comparisons are planned, and all *p* values will be two-sided. A *p* value <0.05 will be considered statistically significant. All statistical analyses will be performed using the SAS software program, version 9.4 (SAS Institute, Cary, NC, USA). The statistical analyses will be described as a priori in a statistical analysis plan.

### Study oversight and organization

The PRIZE study is an investigator-initiated clinical trial conducted by a study organization consisting of the following members (Additional file 1). The principle investigator of the study is Koichi Node, Department of Cardiovascular Medicine, Saga University. A steering committee will be responsible for study design and scientific execution. An executive committee will advise on planning and management of the study. An independent data and safety monitoring board will evaluate safety during the study period. A clinical event committee, blinded to any information related to group allocation, will centrally evaluate the clinical events. Carotid IMT will be measured at a central location, Tsukuba University. Data monitoring will be enforced to ensure the research is performed properly, with an independent audit team inspecting several main hospitals to ensure the quality of the data. Sub-study groups will analyze each exploratory endpoint such as FMD, PWV/CAVI, IMT, echocardiography, and AI.

### Study progress and current status

The PRIZE study was registered by the UMIN in January 2014 (ID: 000012911). End of recruitment period was initially set at January 2016; however, this has been extended to June 2016 due to a shortage of study participants. At present (31 March, 2016), a total of 456 patients have been recruited into the study.

## Discussion

Although hyperuricemia is associated strongly with the MetS, including HT, HL, and type 2 DM, and subsequent risk of atherosclerosis, its significance as an independent risk factor for clinical outcome still remains somewhat controversial [16, 38]. Recently, a retrospective cohort study showed that patients with gout, especially women, had a higher risk of developing CVD after adjustment for vascular risk factors [39]. The prevalence of gout in US adults is about 3.9 %, while the prevalence of hyperuricemia, a precondition necessary for developing gout, is over 21 % [40]. It is therefore essential to prevent patients with asymptomatic hyperuricemia from developing gout by using comprehensive therapeutic strategies. Several studies have shown positive correlations between UA levels and FMD, atherosclerotic plaques, and IMT in the carotid artery [4, 12, 41]. The incidence of hyperuricemia is also high in patients with chronic heart failure and is a predictive factor for prognosis of the condition [42, 43]. Moreover, increased levels of UA cause direct damage to renal tissues, resulting in development of chronic kidney disease [44, 45], and have recently been identified as an independent atherogenic risk factor. There is evidence that excessive production of ROS via UA per sé and UA metabolic processes play a pivotal role in the development of oxidative stress and the pathophysiology of atherosclerosis [46, 47]. ROS promote the oxidation of lipids, making them more atherogenic, and also inactivate nitric oxide (NO). Peroxynitrite is produced by the interaction between NO and the radical superoxide anion, a strong oxidant that contributes to inactivation of proteins [47]. Exposure to ROS changes vascular gene expression, leading to increased inflammatory chemokine production, expression of adhesion molecules, and cellular proliferation and hypertrophy [48]. Despite a strong rationale for the important role of ROS in the development of atherosclerosis, there is still no clinical evidence that scavenging ROS prevents progression of atherosclerosis [49, 50]. A possible explanation for the failure of anti-oxidative therapy may be due, in part, to the highly adaptive responses in the cellular defense system and compartmentalization of ROS signaling. More specific and mechanistic approaches to affect ROS-generating systems and inhibition of vascular oxidative stress are therefore required.

XO is an enzyme responsible for catalyzing the final steps of the metabolic process for purines, specifically the conversion of hypoxanthine to xanthine and subsequently to UA. The degradation of purines to UA is a source of ROS production that has detrimental effects on atherosclerosis progression. Experimental studies have also shown that XO binds to endothelial cells and inactivates NO [51]. Based on this evidence of the physiological role for XO, strategies for XO inhibition are considered reasonable for inhibiting progression of atherosclerosis. However, no large randomized trials have assessed the effect of XO inhibitors on atherosclerosis, although a recent meta-analysis demonstrated that XO inhibition had favorable effects on endothelial function [52].

Febuxostat is a novel selective non-purine XO inhibitor, which has higher affinity for both the oxidized and reduced forms of XO [53]. Due to mechanistic differences in action compared to conventional XO inhibitors, febuxostat has a stronger inhibitory effect on XO [29]. Sezai et al. [27, 54] reported that compared with allopurinol, febuxostat had superior anti-oxidative and anti-atherogenic activities, and renoprotective effect, in addition to a significantly greater UA-lowering effect in post-cardiac surgery patients with hyperuricemia. Febuxostat has been shown to improve endothelial function and suppress atherosclerotic plaque formation by reducing enhanced XO activity in ApoE^(−/−)^ mice [30]. In addition, febuxostat was reported to contribute to cardioprotection through its anti-oxidant and anti-apoptotic effects in doxorubicin-induced cardiomyopathy model rats [55]. The PRIZE study was designed based on these experimental evidences, and the study is considered to be important and meaningful for the clinical implications of febuxostat.

Accumulated evidence indicates that noninvasive assessment of carotid artery IMT is used widely to assess atherosclerosis as a surrogate marker and is associated strongly with an increased risk of cardiovascular disease [56], presence of coronary artery disease, and occurrence of cardiovascular events even after adjustment for known cardiac risk factors [57–61]. Previous studies have reported that UA level is an independent risk factor for carotid IMT in patients with hypertension or type 2 DM. [62, 63]. However, there is only limited evidence whether pharmacological intervention with a XO inhibitor reduces the progression of carotid IMT in patients with asymptomatic hyperuricemia. Recently, Liu et al. [64] reported a randomized single center trial, in which 176 patients with type 2 DM and asymptomatic hyperuricemia were randomized into either allopurinol or conventional treatment groups. The results showed allopurinol not only improved insulin resistance but also reduced carotid IMT, compared to the conventional treatment group. Therefore, XO inhibitors may potentially hinder the progression of carotid atherosclerosis. As mentioned earlier, because febuxostat is more effective for inhibiting XO, a greater inhibitory effect on carotid IMT would also be expected in our study.

It is reported that the levels of UA in women are obviously lower than in men, and generally there is a stronger association between serum UA and cardiovascular events in women than in men [14, 65]. In contrast, Zhao et al. [66] reported that elevated levels of UA were associated with increased risk of all-cause mortality in men, but not in women. In men without the Mets but not in men with the Mets, or in women with or without the Mets, the prevalence of carotid atherosclerosis was similarly associated with increased UA levels [4, 12]. The atherosclerosis risk in communities (ARIC) study, however, demonstrated that serum UA levels per sé might not be a risk factor for carotid atherosclerosis in both genders [67]. In addition, in the randomized clinical trials to evaluate the therapeutic effect of XO inhibitors in patients with gout, gender specific effects of XO inhibitors were not fully evaluated due to the fact that the majority of participants were male [68–70]. Accordingly, the specific gender difference in the contribution of serum UA to the cardiovascular complications and mortality may be, in part, controversial, and the effect of febuxostat on the carotid atherosclerosis still remains to be determined. Because gender is set as an allocation factor in the prize study, the gender specific analyses may be able to address such clinical questions.

In conclusions, on the basis of these backgrounds, we have designed and initiated a randomized multicenter, investigator-initiated trial to test the hypothesis that UA-lowering treatment by the XO inhibitor, febuxostat, for 24 months may delay the progression of carotid IMT in Japanese patients with asymptomatic hyperuricemia. This study may provide important evidence that febuxostat has anti-atherosclerotic actions on carotid IMT and enhance the clinical significance of UA-lowering treatment by febuxostat.

## Additional file

10.1186/s12933-016-0409-2 Trial organization.

## Abbreviations

AI: augmentation index
CAVI: cardio-ankle vascular index
CCA: common carotid artery
CI: confidence interval
CVD: cardiovascular disease
DM: diabetes mellitus
FMD: flow-mediated dilatation
HDL: high-density lipoprotein
HL: hyperlipidemia
HT: hypertension
ICA: internal carotid artery
IMT: intima-media thickness
LDL: low-density lipoprotein
MetS: metabolic syndrome
MMRM: mixed effects models for repeated measures
NO: nitric oxide
PROBE: prospective, randomized, open-label and blinded-endpoint evaluation
PWV: pulse wave velocity
ROS: reactive oxygen species
SD: standard deviation
UA: uric acid
XO: xanthine oxidase

## References

[1] So A, Thorens B (2010). Uric acid transport and disease. J Clin Invest.

[2] Culleton BF, Larson MG, Kannel WB, Levy D (1999). Serum uric acid and risk for cardiovascular disease and death: the Framingham heart study. Ann Intern Med.

[3] Choi HK, Atkinson K, Karlson EW, Curhan G (2005). Obesity, weight change, hypertension, diuretic use, and risk of gout in men: the health professionals follow-up study. Arch Intern Med.

[4] Ishizaka N, Ishizaka Y, Toda E, Nagai R, Yamakado M (2005). Association between serum uric acid, metabolic syndrome, and carotid atherosclerosis in Japanese individuals. Arterioscler Thromb Vasc Biol.

[5] Taniguchi Y, Hayashi T, Tsumura K, Endo G, Fujii S, Okada K (2001). Serum uric acid and the risk for hypertension and Type 2 diabetes in Japanese men: the Osaka Health Survey. J Hypertens.

[6] Milionis HJ, Kakafika AI, Tsouli SG, Athyros VG, Bairaktari ET, Seferiadis KI (2004). Effects of statin treatment on uric acid homeostasis in patients with primary hyperlipidemia. Am Heart J.

[7] Lee J, Sparrow D, Vokonas PS, Landsberg L, Weiss ST (1995). Uric acid and coronary heart disease risk: evidence for a role of uric acid in the obesity-insulin resistance syndrome. The normative aging study. Am J Epidemiol.

[8] Bengtsson C, Lapidus L, Stendahl C, Waldenström J (1988). Hyperuricaemia and risk of cardiovascular disease and overall death. A 12-year follow-up of participants in the population study of women in Gothenburg, Sweden. Acta Med Scand.

[9] Cappuccio FP, Strazzullo P, Farinaro E, Trevisan M (1993). Uric acid metabolism and tubular sodium handling. Results from a population-based study. JAMA.

[10] Meisinger C, Koenig W, Baumert J, Döring A (2008). Uric acid levels are associated with all-cause and cardiovascular disease mortality independent of systemic inflammation in men from the general population: the MONICA/KORA cohort study. Arterioscler Thromb Vasc Biol.

[11] Verdecchia P, Schillaci G, Reboldi G, Santeusanio F, Porcellati C, Brunetti P (2000). Relation between serum uric acid and risk of cardiovascular disease in essential hypertension. The PIUMA study. Hypertension.

[12] Takayama S, Kawamoto R, Kusunoki T, Abe M, Onji M (2012). Uric acid is an independent risk factor for carotid atherosclerosis in a Japanese elderly population without metabolic syndrome. Cardiovasc Diabetol.

[13] Kawamoto R, Tomita H, Oka Y, Ohtsuka N (2006). Relationship between serum uric acid concentration, metabolic syndrome and carotid atherosclerosis. Intern Med.

[14] Fang J, Alderman MH (2000). Serum uric acid and cardiovascular mortality the NHANES I epidemiologic follow-up study, 1971-1992. National Health and Nutrition Examination Survey. JAMA.

[15] Gagliardi AC, Miname MH, Santos RD (2009). Uric acid: a marker of increased cardiovascular risk. Atherosclerosis.

[16] Sakata K, Hashimoto T, Ueshima H, Okayama A (2001). NIPPON DATA 80 Research Group. Absence of an association between serum uric acid and mortality from cardiovascular disease: NIPPON DATA 80, 1980–1994. National integrated projects for prospective observation of non-communicable diseases and its trend in the aged. Eur J Epidemiol.

[17] Bonora E, Targher G, Zenere MB, Saggiani F, Cacciatori V, Tosi F (1996). Relationship of uric acid concentration to cardiovascular risk factors in young men. Role of obesity and central fat distribution. The verona young men atherosclerosis risk factors study. Int J Obes Relat Metab Disord.

[18] Vuorinen-Markkola H, Yki-Järvinen H (1994). Hyperuricemia and insulin resistance. J Clin Endocrinol Metab.

[19] Butler R, Morris AD, Belch JJ, Hill A, Struthers AD (2000). Allopurinol normalizes endothelial dysfunction in type 2 diabetics with mild hypertension. Hypertension.

[20] Doehner W, Schoene N, Rauchhaus M, Leyva-Leon F, Pavitt DV, Reaveley DA (2002). Effects of xanthine oxidase inhibition with allopurinol on endothelial function and peripheral blood flow in hyperuricemic patients with chronic heart failure: results from 2 placebo-controlled studies. Circulation.

[21] Leyva F, Anker S, Swan JW, Godsland IF, Wingrove CS, Chua TP (1997). Serum uric acid as an index of impaired oxidative metabolism in chronic heart failure. Eur Heart J.

[22] Mustard JF, Murphy EA, Ogryzlo MA, Smythe HA (1963). Blood coagulation and platelet economy in subjects with primary gout. Can Med Assoc J.

[23] Nakagawa T, Hu H, Zharikov S, Tuttle KR, Short RA, Glushakova O (2006). A causal role for uric acid in fructose-induced metabolic syndrome. Am J Physiol Renal Physiol.

[24] Kamatani N, Fujimori S, Hada T, Hosoya T, Kohri K, Nakamura T (2011). An allopurinol-controlled, randomized, double-dummy, double-blind, parallel between-group, comparative study of febuxostat (TMX-67), a non-purine-selective inhibitor of xanthine oxidase, in patients with hyperuricemia including those with gout in Japan: phase 3 clinical study. J Clin Rheumatol.

[25] Hatoum H, Khanna D, Lin SJ, Akhras KS, Shiozawa A, Khanna P (2014). Achieving serum urate goal: a comparative effectiveness study between allopurinol and febuxostat. Postgrad Med.

[26] Faruque LI, Ehteshami-Afshar A, Wiebe N, Tjosvold L, Homik J, Tonelli M (2013). A systematic review and meta-analysis on the safety and efficacy of febuxostat versus allopurinol in chronic gout. Semin Arthritis Rheum.

[27] Sezai A, Soma M, Nakata K, Hata M, Yoshitake I, Wakui S (2013). Comparison of febuxostat and allopurinol for hyperuricemia in cardiac surgery patients (NU-FLASH Trial). Circ J.

[28] Malik UZ, Hundley NJ, Romero G, Radi R, Freeman BA, Tarpey MM (2011). Febuxostat inhibition of endothelial-bound XO: implications for targeting vascular ROS production. Free Radic Biol Med.

[29] Okamoto K, Eger BT, Nishino T, Kondo S, Pai EF, Nishino T (2003). An extremely potent inhibitor of xanthine oxidoreductase. Crystal structure of the enzyme-inhibitor complex and mechanism of inhibition. J Biol Chem.

[30] Nomura J, Busso N, Ives A, Matsui C, Tsujimoto S, Shirakura T (2014). Xanthine oxidase inhibition by febuxostat attenuates experimental atherosclerosis in mice. Sci Rep.

[31] Japanese Society of Gout and Nucleic Acid Metabolism (2012). Guideline for the management of hyperuricemia and gout.

[32] Brown S, Thorpe H, Hawkins K, Brown J (2005). Minimization–reducing predictability for multi-centre trials whilst retaining balance within centre. Stat Med.

[33] Stein JH, Korcarz CE, Hurst RT, Lonn E, Kendall CB, Mohler ER (2008). Use of carotid ultrasound to identify subclinical vascular disease and evaluate cardiovascular disease risk: a consensus statement from the American Society of Echocardiography Carotid Intima-Media Thickness Task Force. Endorsed by the Society for Vascular Medicine. J Am Soc Echocardiogr.

[34] Touboul PJ, Hennerici MG, Meairs S, Adams H, Amarenco P, Desvarieux M (2004). Mannheim intima-media thickness consensus. Cerebrovasc Dis.

[35] Sabetai MM, Tegos TJ, Nicolaides AN, Dhanjil S, Pare GJ, Stevens JM (2000). Reproducibility of computer-quantified carotid plaque echogenicity: can we overcome the subjectivity?. Stroke.

[36] Lundby-Christensen L, Almdal TP, Carstensen B, Tarnow L, Wiinberg N (2010). Carotid intima-media thickness in individuals with and without type 2 diabetes: a reproducibility study. Cardiovasc Diabetol.

[37] Ikeda K, Takahashi T, Yamada H, Matsui K, Sawada T, Nakamura T (2013). Effect of intensive statin therapy on regression of carotid intima-media thickness in patients with subclinical carotid atherosclerosis (a prospective, randomized trial: PEACE (pitavastatin evaluation of atherosclerosis regression by intensive cholesterol-lowering therapy) study). Eur J Prev Cardiol.

[38] Feig DI, Mazzali M, Kang DH, Nakagawa T, Price K, Kannelis J (2006). Serum uric acid: a risk factor and a target for treatment?. J Am Soc Nephrol.

[39] Clarson LE, Hider SL, Belcher J, Heneghan C, Roddy E, Mallen CD (2015). Increased risk of vascular disease associated with gout: a retrospective, matched cohort study in the UK clinical practice research datalink. Ann Rheum Dis.

[40] Zhu Y, Pandya BJ, Choi HK (2011). Prevalence of gout and hyperuricemia in the US general population: the National Health and Nutrition Examination Survey 2007–2008. Arthritis Rheum.

[41] Tomiyama H, Higashi Y, Takase B, Node K, Sata M, Inoue T (2011). Relationships among hyperuricemia, metabolic syndrome, and endothelial function. Am J Hypertens.

[42] Anker SD, Doehner W, Rauchhaus M, Sharma R, Francis D, Knosalla C (2003). Uric acid and survival in chronic heart failure: validation and application in metabolic, functional, and hemodynamic staging. Circulation.

[43] Hamaguchi S, Furumoto T, Tsuchihashi-Makaya M, Goto K, Goto D, Yokota T (2011). Hyperuricemia predicts adverse outcomes in patients with heart failure. Int J Cardiol.

[44] Iseki K, Ikemiya Y, Inoue T, Iseki C, Kinjo K, Takishita S (2004). Significance of hyperuricemia as a risk factor for developing ESRD in a screened cohort. Am J Kidney Dis.

[45] Zhu P, Liu Y, Han L, Xu G, Ran JM (2014). Serum uric acid is associated with incident chronic kidney disease in middle-aged populations: a meta-analysis of 15 cohort studies. PLoS One.

[46] Puddu P, Puddu GM, Cravero E, Vizioli L, Muscari A (2012). Relationships among hyperuricemia, endothelial dysfunction and cardiovascular disease: molecular mechanisms and clinical implications. J Cardiol.

[47] Stocker R, Keaney JF (2004). Role of oxidative modifications in atherosclerosis. Physiol Rev.

[48] Taniyama Y, Griendling KK (2003). Reactive oxygen species in the vasculature: molecular and cellular mechanisms. Hypertension.

[49] Hasnain BI, Mooradian AD (2004). Recent trials of antioxidant therapy: what should we be telling our patients?. Cleve Clin J Med.

[50] Morris CD, Carson S (2003). Routine vitamin supplementation to prevent cardiovascular disease: a summary of the evidence for the US preventive services task force. Ann Intern Med.

[51] Houston M, Estevez A, Chumley P, Aslan M, Marklund S, Parks DA (1999). Binding of xanthine oxidase to vascular endothelium. Kinetic characterization and oxidative impairment of nitric oxide-dependent signaling. J Biol Chem.

[52] Higgins P, Dawson J, Lees KR, McArthur K, Quinn TJ, Walters MR (2012). Xanthine oxidase inhibition for the treatment of cardiovascular disease: a systematic review and meta-analysis. Cardiovasc Ther.

[53] Takano Y, Hase-Aoki K, Horiuchi H, Zhao L, Kasahara Y, Kondo S (2005). Selectivity of febuxostat, a novel non-purine inhibitor of xanthine oxidase/xanthine dehydrogenase. Life Sci.

[54] Sezai A, Soma M, Nakata K, Osaka S, Ishii Y, Yaoita H (2015). Comparison of febuxostat and allopurinol for hyperuricemia in cardiac surgery patients with chronic kidney disease (NU-FLASH trial for CKD). J Cardiol.

[55] Krishnamurthy B, Rani N, Bharti S, Golechha M, Bhatia J, Nag TC (2015). Febuxostat ameliorates doxorubicin-induced cardiotoxicity in rats. Chem Biol Interact.

[56] Davis PH, Dawson JD, Riley WA, Lauer RM (2001). Carotid intimal-medial thickness is related to cardiovascular risk factors measured from childhood through middle age: the Muscatine Study. Circulation.

[57] Chambless LE, Heiss G, Folsom AR, Rosamond W, Szklo M, Sharrett AR (1997). Association of coronary heart disease incidence with carotid arterial wall thickness and major risk factors: the atherosclerosis risk in communities (ARIC) study, 1987–1993. Am J Epidemiol.

[58] Craven TE, Ryu JE, Espeland MA, Kahl FR, McKinney WM, Toole JF (1990). Evaluation of the associations between carotid artery atherosclerosis and coronary artery stenosis. A case-control study. Circulation.

[59] Handa N, Matsumoto M, Maeda H, Hougaku H, Ogawa S, Fukunaga R (1990). Ultrasonic evaluation of early carotid atherosclerosis. Stroke.

[60] Khoury Z, Schwartz R, Gottlieb S, Chenzbraun A, Stern S, Keren A (1997). Relation of coronary artery disease to atherosclerotic disease in the aorta, carotid, and femoral arteries evaluated by ultrasound. Am J Cardiol.

[61] Salonen JT, Salonen R (1993). Ultrasound B-mode imaging in observational studies of atherosclerotic progression. Circulation.

[62] Tavil Y, Kaya MG, Oktar SO, Sen N, Okyay K, Yazici HU (2008). Uric acid level and its association with carotid intima-media thickness in patients with hypertension. Atherosclerosis.

[63] Li Q, Yang Z, Lu B, Wen J, Ye Z, Chen L (2011). Serum uric acid level and its association with metabolic syndrome and carotid atherosclerosis in patients with type 2 diabetes. Cardiovasc Diabetol.

[64] Liu P, Wang H, Zhang F, Chen Y, Wang D, Wang Y (2015). The effects of allopurinol on the carotid intima-media thickness in patients with type 2 diabetes and asymptomatic hyperuricemia: a 3-year randomized parallel-controlled study. Intern Med.

[65] Freedman DS, Williamson DF, Gunter EW, Byers T (1995). Relation of serum uric acid to mortality and ischemic heart disease. The NHANES I epidemiologic follow-up study. Am J Epidemiol.

[66] Zhao G, Huang L, Song M, Song Y (2013). Baseline serum uric acid level as a predictor of cardiovascular disease related mortality and all-cause mortality: a meta-analysis of prospective studies. Atherosclerosis.

[67] Iribarren C, Folsom AR, Eckfeldt JH, McGovern PG, Nieto FJ (1996). Correlates of uric acid and its association with asymptomatic carotid atherosclerosis: the ARIC study. Atherosclerosis risk in communities. Ann Epidemiol.

[68] Becker MA, Schumacher HR, Wortmann RL, MacDonald PA, Eustace D, Palo WA (2005). Febuxostat compared with allopurinol in patients with hyperuricemia and gout. N Engl J Med.

[69] Schumacher HR, Becker MA, Wortmann RL, Macdonald PA, Hunt B, Streit J (2008). Effects of febuxostat versus allopurinol and placebo in reducing serum urate in subjects with hyperuricemia and gout: a 28-week, phase III, randomized, double-blind, parallel-group trial. Arthritis Rheum.

[70] Huang X, Du H, Gu J, Zhao D, Jiang L, Li X (2014). An allopurinol-controlled, multicenter, randomized, double-blind, parallel between-group, comparative study of febuxostat in Chinese patients with gout and hyperuricemia. Int J Rheum Dis.

